# Fontana’s Stain of Spirochaete Pallida in Films

**Published:** 1914-10

**Authors:** 


**Fontana's Stain of Spirochaete Pallida in Films.** Col. Birt, Jour. of Royal Army Med. Corps, Mch., 1914, considers this superior to the India ink or ordinary silver method, in dry smears. Films may be fixed by drying in air but must not be heated. They are moistened with Huge's solution (Acetic acid, 1%; formalin 20% in water), poured over them several times in a minute. They are washed in water and treated with a mordant consisting of 5% of tannic acid in 1% phenol solution. The slide, covered with the mordant is heated till it steams, left for 30 seconds and then washed with water for 15-30 seconds. Without drying, the stain consisting of 1% silver nitrate plus a trace of ammonia to produce turbidity, not enough to redissolve, is poured on the slide which is again heated to steaming point and left for 30 seconds. It is then washed with water, dried with blotting paper and mounted in xylol balsam permanently. Fading soon occurs if cedar oil is used. The spirocraetes stands out jet-black in a clear field appearing thicker than when stained with aniline dyes. They can, therefore, be detected promptly.

